# Dual effects of noradrenaline on astroglial production of chemokines and pro-inflammatory mediators

**DOI:** 10.1186/1742-2094-10-81

**Published:** 2013-07-09

**Authors:** Ara E Hinojosa, Javier R Caso, Borja García-Bueno, Juan C Leza, José LM Madrigal

**Affiliations:** 1 Departamento de Farmacología, Facultad de Medicina, Universidad Complutense de Madrid (UCM), Centro de Investigación Biomédica en Red de Salud Mental (CIBERSAM), Instituto de Investigación Hospital 12 de Octubre (Imas12), Instituto de Investigación en Neuroquímica UCM, Avenida Complutense s/n, Madrid 28040, Spain; 2 Departamento de Psiquiatría, Facultad de Medicina, Universidad Complutense de Madrid (UCM), Centro de Investigación Biomédica en Red de Salud Mental (CIBERSAM), Instituto de Investigación Hospital 12 de Octubre (Imas12), Instituto de Investigación en Neuroquímica UCM, Avenida Complutense s/n, Madrid 28040, Spain

**Keywords:** Fractalkine, CX3CL1, MCP-1, CCL2, Astrocytes, Inflammation

## Abstract

**Background:**

Noradrenaline (NA) is known to limit neuroinflammation. However, the previously described induction by NA of a chemokine involved in the progression of immune/inflammatory processes, such as chemokine (C-C motif) ligand 2 (CCL2)/monocyte chemotactic protein-1 (MCP-1), apparently contradicts NA anti-inflammatory actions. In the current study we analyzed NA regulation of astroglial chemokine (C-X3-C motif) ligand 1 (CX3CL1), also known as fractalkine, another chemokine to which both neuroprotective and neurodegenerative actions have been attributed. In addition, NA effects on other chemokines and pro-inflammatory mediators were also analyzed.

**Methods:**

Primary astrocyte-enriched cultures were obtained from neonatal Wistar rats. These cells were incubated for different time durations with combinations of NA and lipopolysaccharide (LPS). The expression and synthesis of different proteins was measured by RT-PCR and enzyme-linked immunosorbent assay (ELISA) or enzyme immunoassays. Data were analyzed by one-way analysis of variance (ANOVA), followed by Newman-Keuls multiple comparison tests.

**Results:**

The data presented here show that in control conditions, NA induces the production of CX3CL1 in rat cultured astrocytes, but in the presence of an inflammatory stimulus, such as LPS, NA has the opposite effect inhibiting CX3CL1 production. This inversion of NA effect was also observed for MCP-1. Based on the observation of this dual action, NA regulation of different chemokines and pro-inflammatory cytokines was also analyzed, observing that in most cases NA exerts an inhibitory effect in the presence of LPS. One characteristic exception was the induction of cyclooxygenase-2 (COX-2), where a summative effect was detected for both LPS and NA.

**Conclusion:**

These data suggest that NA effects on astrocytes can adapt to the presence of an inflammatory agent reducing the production of certain cytokines, while in basal conditions NA may have the opposite effect and help to maintain moderate levels of these cytokines.

## Background

Noradrenaline (NA) is recognized as a key modulator in the regulation of multiple central nervous system (CNS) activities, such as mood changes [1], memory formation [2], stress response [3] and cellular energy metabolism [4], among others. However, in relation to the study of mechanisms involved in the development of diseases with a neurological component, the main attribute of NA is its ability to reduce the neuroinflammatory processes associated to them [5]. The degeneration of the main noradrenergic area in the brain, the locus coeruleus, seems to be one of the stages preceding the subsequent development of neuronal death observed in Alzheimer’s disease [6]. In fact, diverse experimental settings indicate that NA interactions with different types of brain cells lead to the regulation of inflammatory pathways and mediators [5].

Based on this, a sound hypothesis proposes that the loss of NA constitutive levels can create a ‘permissive’ environment for the development of inflammation and subsequent neurodegeneration [7].

Among other roles, astrocytes are in charge of the surveillance of the CNS status, providing alarm signals when threat is detected and helping to maintain CNS homeostasis [8]. Thus, we have focused our work on the analysis of NA interaction with astrocytes, to help elucidate the means through which NA protects neurons against different types of injuries.

We previously observed that NA, through the activation of β2-adrenergic receptors and the elevation of cAMP, induces the production of the chemokine (C-C motif) ligand 2 (CCL2)/monocyte chemotactic protein 1 (MCP-1) and protects neurons against excitotoxicity [9]. This fact, while contradictory with the well-known actions of CCL2 as a chemoattractant that facilitate the progression of the immune and inflammatory responses (which can have fatal consequences for nearby cells) [10], is in agreement with several studies which describe neuroprotective actions of CCL2 against multiple types of injuries [11]. This observation led us to the analysis of NA regulation of different chemokines on astrocytes.

As their name suggests, all chemokines can attract those cells expressing the specific receptors. This explains their involvement in very disparate processes where cell migration is present, such as cell adhesion/trafficking [12], angiogenesis [13] or progenitor cell migration [14]. In addition, as mentioned, chemokines by themselves may also cause some changes in cell functioning by direct interaction with such cells. In fact, new actions unrelated to the regulation of cell migration have been recently discovered for chemokines, highlighting the potential relevance of this family of proteins, particularly in the field of neuroinflammation [11].

One of the chemokines that has proven to play a significant role in the regulation of brain physiology is chemokine (C-X3-C motif) ligand 1 (CX3CL1), also known as fractalkine or neurotactin. CX3CL1 is expressed by neurons, astrocytes and microglia, and its specific receptor CX3CR1 is also expressed by all these cell types [15]. However, since the main production of CX3CL1 is observed in neurons and the receptor seems to be more abundant in glial cells, it has been proposed that CX3CL1 serves as an intermediary used by neurons to communicate with glial cells. While several studies have shown that CX3CL1 can modulate neuronal activity and survival, others indicate that the restrain of CX3CL1 activity can also prevent neuronal damage in certain pathologies [16-18]. This potential dual role of CX3CL1 has been proposed to be dependent on the different stages of certain neurodegenerative diseases where microglia activation may be beneficial (by their ability to remove apoptotic cells and toxic debris) or detrimental for neurons (by the elimination of healthy cells) [19].

The present study describes the induction of CX3CL1 expression and synthesis by NA in astrocytes. The results obtained also indicate that in the presence of a pro-inflammatory stimulus, such as lipopolysaccharide (LPS) from gram-negative bacteria, which causes a large production of CX3CL1 by astrocytes, NA has the opposite effect inhibiting CX3CL1 production. This observation led us to analyze if this also applies to CCL2 and other related chemokines, as well as different pro-inflammatory mediators. The data show that while each one of the proteins evaluated has a different regulation by NA, in most cases where NA induced their expression in control conditions, the presence of LPS switched NA effect towards inhibition. This suggests that, in the brain, NA may be responsible for the maintenance of the constitutive levels of certain factors, while it can repress the overproduction in inflammatory situations.

## Methods

### Reagents

Fetal calf serum (<10 EU/ml) and Dulbecco’s modified Eagle’s medium (DMEM) were obtained from Gibco Life Technologies (Carlsbad, CA, USA). LPS from *Escherichia coli* 0111:B4 and NA for cell treatments, and glutamine, penicillin and streptomycin for cell cultures, were obtained from Sigma-Aldrich (St Louis, MO, USA). TRIzol, *Taq* polymerase and cDNA synthesis reagents were obtained from Invitrogen (Carlsbad, CA, USA).

### Astrocyte cultures

All experimental protocols adhered to the guidelines of the Animal Welfare Committee of the Universidad Complutense of Madrid, Spain, and according to European Union laws. Rat cortical astrocytes were obtained as previously described [20]. Briefly, 1-day-old Wistar rats (Harlan, Indianapolis, IN, USA) were used to prepare primary mixed glial cultures. Microglia were detached by gentle shaking after 11 to 13 days in culture. Astrocytes were prepared by mild trypsinization of the remaining cells, replated at 6 × 10^5^ cells/ml, and consisted of 95% astrocytes as determined by staining for glial fibrillary acidic protein (GFAP) and <5% microglial as determined by staining with the specific marker OX-42.

### mRNA analysis

Total cytoplasmic RNA was prepared from cells using TRIzol reagent and aliquots were converted to cDNA using random hexamer primers. Quantitative changes in mRNA levels were estimated by real-time PCR (qPCR) using the following cycling conditions: 35 cycles of denaturation at 95°C for 10 seconds, annealing at 58 to 61°C for 15 seconds, depending on the specific set of primers, and extension at 72°C for 20 seconds. Reactions were carried out in the presence of SYBR green (1:10,000 dilution of stock solution from Molecular Probes, Eugene, OR, USA) and in a 20 μl reaction in Rotor-Gene (Corbett Research, Mortlake, Australia). Primers for the genes of interest were designed based on the rat sequences deposited in GenBank (Table 1). Relative mRNA concentrations were calculated from the take-off point of reactions using included software, and glyceraldehyde 3-phosphate dehydrogenase (GAPDH) levels used to normalize data.

**Table 1:** Primers used for RT-PCR

**Gene name**	**Forward primer**	**Reverse primer**
CX3CL1	5′-AATCCCAGTGACCTTGCTCATCCA	5′-TGGACCCATTTCTCCTTTGGGTCA
CCL2	5′-TGCTGTCTCAGCCAGATGCAGTTA	5′-TACAGCTTCTTTGGGACACCTGCT
CCL6	5′-TGTTCCAGCAGGGCATCTTCTTCT	5′-GCCTCATTTGCATGGAGAGCCATT
CCL7	5′-GGACCAATTCATCCACTTGCTGCT	5′-TCTGATGGGCTTCAGCACAGACTT
CCL12	5′-TGAGTCCTCCAGCTCTCATTCCAA	5′-TGAACACTGAATCTGGTCCAGCCA
CXCL16	5′-TGTCGCTGGAAGTTGCTACTGTGA	5′-TCTTGGACTGCAACTGGAACCTGA
IL-1β	5′-ACCTGCTAGTGTGTGATGTTCCCA	5′-AGGTGGAGAGCTTTCAGCTCACAT
TNFα	5′-CTGGCCAATGGCATGGATCTCAAA	5′-AGCCTTGTCCCTTGAAGAGAACCT
IFNγ	5′-AAAGACAACCAGGCCATCAGCAAC	5′-TCTGTGGGTTGTTCACCTCGAACT
COX-2	5′-GCATTCTTTGCCCAGCACTTCACT	5′-TTTAAGTCCACTCCATGGCCCAGT
GAPDH	5′-TGCACCACCAACTGCTTAGC	5′-GGCATGGACTGTGGTCATGAG

*CCL12* chemokine (C-C motif) ligand 12, *CCL2* chemokine (C-C motif) ligand 2, *CCL6* chemokine (C-C motif) ligand 6, *CCL7* chemokine (C-C motif) ligand 7, *COX-2* cyclooxygenase-2, *CX3CL1* chemokine (C-X3-C motif) ligand 1, *CXCL16* chemokine (C-X-C motif) ligand 16, *GAPDH* glyceraldehyde 3-phosphate dehydrogenase, *IFNγ* interferon gamma, *IL-1β* interleukin-1 beta, *TNFα* tumor necrosis factor alpha.

### CCL2, CX3CL1, CCL6 and TNFα measurement

Protein levels in the incubation medium were detected using specific enzyme-linked immunosorbent assay (ELISA), carried out according to the manufacturer’s instructions: R&D Systems Inc (Minneapolis, MN, USA) for CX3CL1; BD Biosciences (San Jose, CA, USA) for CCL2; CUSABIO (Wuhan, China) for chemokine (C-C motif) ligand 6 (CCL6); and RayBiotech (Atlanta, GA, USA) for tumor necrosis factor alpha (TNFα). Briefly, the medium was collected from the astrocyte cultures and stored at −80°C until the day of the assay (avoiding repeated freeze-thaw cycles). A standard curve was generated using the standards provided in the kits. The assay detection limits were: 31.3 to 2,000 pg/ml for CCL2; 195 to 12,500 pg/ml for CX3CL1; 0.156 to 10 ng/m for CCL6; and 25 to 20,000 pg/ml for TNFα.

### PGE_2_ measurement

Prostaglandin E_2_ (PGE_2_) levels in the incubation medium were measured using a specific enzyme immunoassay (EIA), carried out according to the manufacturer’s instructions (Cayman Chemical, Ann Arbor, MI, USA). Briefly, the medium was collected from the astrocyte cultures and stored at −80°C until the day of the assay (avoiding repeated freeze-thaw cycles). A standard curve was generated using the rat PGE_2_ standard provided in the kit. The assay detection limit was 15 pg/ml.

### Data analysis

All experiments were undertaken at least in triplicate. Data were analyzed by one-way analysis of variance (ANOVA), followed by Newman-Keuls multiple comparison tests. *P* values <0.05 were considered significant.

## Results

### NA induces CX3CL1 synthesis and release in astrocytes

**Figure 1:**
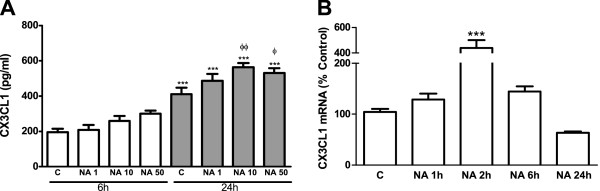
**NA induces CX3CL1 production by astrocytes. (A)** Astrocytes were incubated with control media or NA (1, 10 or 50 μM) for 6 or 24 hours. CX3CL1 levels in the media were assessed by ELISA. ***P <0.001 versus 6-hour control; ^Φ^*P* <0.05 versus 24-hour control; ^ΦΦ^*P* <0.01 versus 24-hour control. Data are means ± SE of n = 12 replicates per group. **(B)** Astrocytes were incubated with control media or NA 10 μM for 1, 2, 6 or 24 hours. RNA was isolated and CX3CL1 mRNA levels determined by RT-PCR. Data are expressed as percentage of control values (set to 100%).***P <0.001 versus control. Data are means ± SE of n = 8 replicates per group. C, control; CX3CL1, chemokine (C-X3-C motif) ligand 1; ELISA, enzyme-linked immunosorbent assay; NA, noradrenaline; RT-PCR, reverse transcription polymerase chain reaction; SE, standard error.

An ELISA assay was used to evaluate the production of CX3CL1 and its release from cultured astrocytes. Different concentrations of NA (1 to 50 μM) were added to the culture medium and the cells were incubated for 6 or 24 hours. Six hours of treatment did not yield significant changes in the concentration of CX3CL1. However, when the incubation period was extended to 24 hours, NA treatment caused an increase with significant differences with respect to the 24-hour control group for concentrations above 10 μM. The concentration of 50 μM did not cause a production of CX3CL1 larger than that observed for 10 μM, suggesting that the amount measured represents NA maximal effect. Interestingly, the control values detected after 24 hours were higher than those detected after 6 hours (Figure 1A), confirming that CX3CL1 is constitutively released by astrocytes at considerable amounts.

Having found that 10 μM is the lowest concentration of NA able to induce a significant induction of CX3CL1, we treated astrocytes for 1 to 24 hours with this amount of NA and used real-time RT-PCR (qRT-PCR) to assess mRNA levels of CX3CL1. This allowed us to observe an elevation that was maximal after 2 hours of incubation. Twenty-four hours after the onset of this treatment, the mRNA levels were lower than in the control group (Figure 1B).

### NA inhibits CX3CL1 production in the presence of LPS in astrocytes

**Figure 2:**
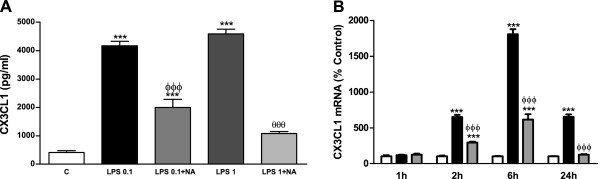
**In the presence of LPS NA inhibits CX3CL1 production by astrocytes. (A)** Astrocytes were incubated with control media, LPS 0.1 and 1 μg/ml alone or in combination with NA 10 μM for 24 hours. CX3CL1 levels in the media were assessed by ELISA. ***P <0.001 versus control; ^ΦΦΦ^*P* <0.001 versus LPS 0.1 μg/ml; ^θθθ^*P* <0.001 versus LPS 1 μg/ml. Data are means ± SE of n = 12 replicates per group. **(B)** Astrocytes were incubated with control media (white columns), LPS 0.1 μg/ml (black columns) or LPS and NA 10 μM (gray columns) for 1, 2, 6 or 24 hours. RNA was isolated and CX3CL1 mRNA levels determined by RT-PCR. Data are expressed as percentage of control values (set to 100%). ***P <0.001 versus control; ^ΦΦΦ^*P* <0.001 versus LPS. Data are means ± SE of n = 8 replicates per group. C, control; CX3CL1, chemokine (C-X3-C motif) ligand 1; ELISA, enzyme-linked immunosorbent assay; LPS, lipopolysaccharide; NA, noradrenaline; RT-PCR, reverse transcription polymerase chain reaction; SE, standard error.

In order to evaluate the magnitude of NA effect, astrocytes were treated with an inflammatory stimulus known to induce CX3CL1 expression in the brain, such as LPS [21]. The incubation with LPS 0.1 μg/ml for 24 hours caused a greater than tenfold elevation of CX3CL1 levels in the culture media as assessed by ELISA (Figure 2A). This indicates that the elevation of CX3CL1 production caused by NA is only minor in relation to the full potential of these cells.

Having previously found the ability of NA to induce CX3CL1 by itself, we decided to analyze its possible interaction with LPS. We observed that it reduces the production of CX3CL1 caused by LPS (Figure 2A) even when a tenfold higher concentration of this endotoxin is used.

Parallel changes were found for CX3CL1 mRNA where LPS caused a large increase that was maximal after 6 hours of treatment and still remained elevated after 24 hours, and NA inhibited LPS effect on CX3CL1 mRNA reducing it to control levels after 24 hours of co-incubation (Figure 2B).

### NA inhibits CCL2 production in the presence of LPS in astrocytes

**Figure 3:**
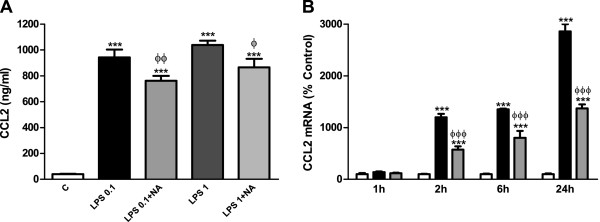
**NA inhibits CCL2 production by astrocytes in the presence of LPS. (A)** Astrocytes were incubated with control media, LPS 0.1 and 1 μg/ml alone or in combination with NA 10 μM for 24 hours. CCL2 levels in the media were assessed by ELISA. ***P <0.001 versus control; ^Φ^*P* <0.05 versus LPS 1 μg/ml; ^ΦΦ^*P* <0.01 versus LPS 0.1 μg/ml. Data are means ± SE of n = 12 replicates per group. **(B)** Astrocytes were incubated with control media (white columns), LPS 0.1 μg/ml (black columns) or LPS and NA 10 μM (gray columns) for 1, 2, 6 or 24 hours. RNA was isolated and CCL2 mRNA levels determined by RT-PCR. Data are expressed as percentage of control values (set to 100%). ***P <0.001 versus control; ^ΦΦΦ^*P* <0.001 versus LPS. Data are means ± SE of n = 8 replicates per group. C, control; CCL2, chemokine (C-C motif) ligand 2; ELISA, enzyme-linked immunosorbent assay; LPS, lipopolysaccharide; NA, noradrenaline; RT-PCR, reverse transcription polymerase chain reaction; SE, standard error.

Since NA presented this double effect for CX3CL1 expression, we tested if it would also have a similar effect for CCL2, another chemokine we previously found that can be induced by NA in astrocytes [9]. ELISA and qRT-PCR studies allowed us to detect a similar pattern of regulation, where LPS caused a large induction of CCL2 that was in part prevented by the co-treatment with NA (Figure 3).

### Alterations in NA regulation of different chemokines

While each chemokine has particular features that differentiate it from the rest, they all have other features in common, and the dual regulation by NA could be one of the characteristics that apply to all of them. Due to the large number of chemokines known to date, a preliminary approach was made by analyzing mRNA regulation in three chemokines with effects similar to those of CCL2 and that share the C-C chemokine receptor type 2 (CCR2) with CCL2, such as CCL6/C10, chemokine (C-C motif) ligand 7 (CCL7)/monocyte chemotactic protein-3 (MCP-3) and chemokine (C-C motif) ligand 12 (CCL12)/monocyte chemotactic protein-5 (MCP-5) [22]. Chemokine (C-X-C motif) ligand 16 (CXCL16)/small inducible cytokine subfamily B member 16 (SCYB16) was also studied because it has also been characterized as a neuroprotective agent that modulates astroglial production of CCL2 [23].

**Figure 4:**
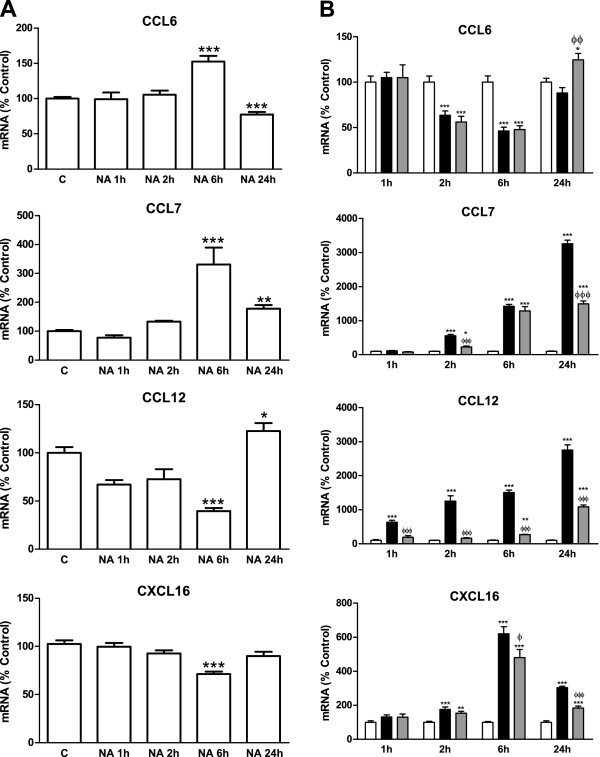
**NA effects on different chemokines. (A)** Astrocytes were incubated with control media or NA 10 μM for 1, 2, 6 or 24 hours. RNA was isolated and mRNA levels of CCL6, CCL7, CCL12 and CXCL16 were determined by RT-PCR. Data are expressed as percentage of control values (set to 100%). *P <0.05 versus control; **P <0.01 versus control; ***P <0.001 versus control. Data are means ± SE of n = 8 replicates per group. **(B)** Astrocytes were incubated with control media (white columns), LPS 0.1 μg/ml (black columns) or LPS and NA 10 μM (gray columns) for 1, 2, 6 or 24 hours. RNA was isolated and mRNA levels of CCL6, CCL7, CCL12 and CXCL16 were determined by RT-PCR. Data are expressed as percentage of control values (set to 100%). *P <0.05 versus control; **P <0.01 versus control; ***P <0.001 versus control; ^Φ^*P* <0.05 versus LPS; ^ΦΦ^*P* <0.01 versus LPS; ^ΦΦΦ^*P* <0.001 versus LPS. Data are means ± SE of n = 8 replicates per group. C, control; CCL6, chemokine (C-C motif) ligand 6; CCL7, chemokine (C-C motif) ligand 7; CCL12, chemokine (C-C motif) ligand 12; CXCL16, chemokine (C-X-C motif) ligand 16; LPS, lipopolysaccharide; NA, noradrenaline; RT-PCR; reverse transcription polymerase chain reaction; SE, standard error.

RT-PCR measurements showed that while 6 hours of incubation with NA elevated CCL6 and CCL7 mRNA concentrations, the opposite effect happened for the other two chemokines analyzed (Figure 4A).

LPS caused an induction of CCL7 larger than the one caused by NA. It also induced CCL12 and CXCL16. For these three chemokines, NA showed an inhibitory effect in the presence of LPS similar to the one observed for CCL2 and CX3CL1 (Figure 4B).

**Figure 5:**
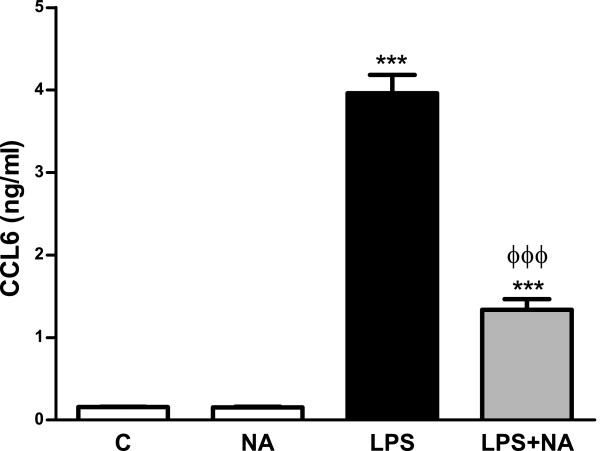
**In the presence of LPS NA inhibits CCL6 production by astrocytes.** Astrocytes were incubated with control media, NA 10 μM or LPS 0.1 μg/ml alone or in combination with NA 10 μM for 24 hours. CCL6 levels in the media were assessed by ELISA. ***P <0.001 versus control; ^ΦΦΦ^*P* <0.001 versus LPS. Data are means ± SE of n = 12 replicates per group. C, control; CCL6, chemokine (C-C motif) ligand 6; ELISA, enzyme-linked immunosorbent assay; LPS, lipopolysaccharide; NA, noradrenaline; SE, standard error.

In order to further analyze our results, ELISA studies were performed for these four chemokines; however, detectable amounts were only found for CCL6. LPS treatment caused an increase in the accumulation of CCL6 that was prevented by NA, while no modifications were caused by NA alone (Figure 5).

### Dual effect of NA on pro-inflammatory mediators

**Figure 6:**
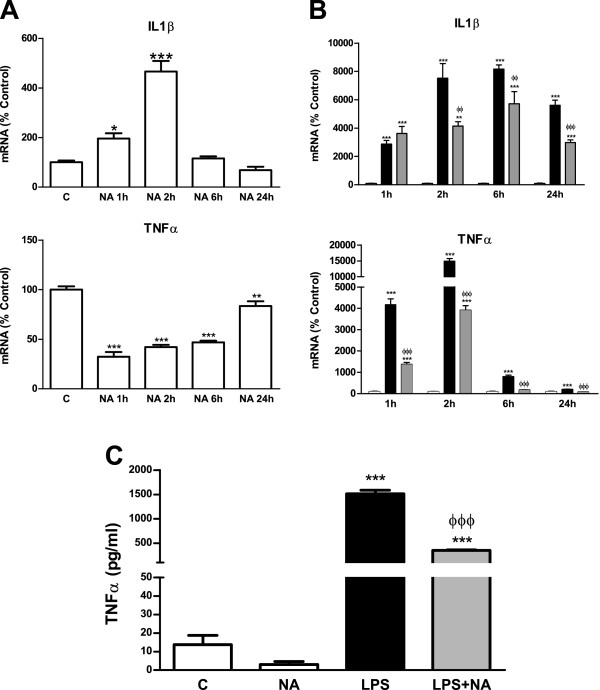
**NA effects on different cytokines. (A)** Astrocytes were incubated with control media or NA 10 μM for 1, 2, 6 or 24 hours. RNA was isolated and mRNA levels of IL-1β, TNFα and IFNγ were determined by RT-PCR. Data are expressed as percentage of control values (set to 100%). *P <0.05 versus control; **P <0.01 versus control; ***P <0.001 versus control. Data are means ± SE of n = 8 replicates per group. **(B)** Astrocytes were incubated with control media (white columns), LPS 0.1 μg/ml (black columns) or LPS and NA 10 μM (gray columns) for 1, 2, 6 or 24 hours. RNA was isolated and mRNA levels of IL-1β, TNFα and IFNγ were determined by RT-PCR. Data are expressed as percentage of control values (set to 100%). **P <0.01 versus control; ***P <0.001 versus control; ^ΦΦ^*P* <0.01 versus LPS; ^ΦΦΦ^*P* <0.001 versus LPS. Data are means ± SE of n = 8 replicates per group. **(C)** Astrocytes were incubated with control media, NA 10 μM, LPS 0.1 μg/ml alone or in combination with NA 10 μM for 24 hours. TNFα levels in the media were assessed by ELISA. ***P <0.001 versus control, ^ΦΦΦ^*P* <0.001 versus LPS. Data are means ± SE of n = 12 replicates per group. C, control; ELISA, enzyme-linked immunosorbent assay; IFNγ, interferon gamma; IL-1β, interleukin-1 beta; LPS, lipopolysaccharide; NA, noradrenaline; RT-PCR, reverse transcription polymerase chain reaction; SE, standard error; TNFα, tumor necrosis factor alpha.

According to our hypothesis, NA may participate in regulating the levels of certain mediators used by brain cells to communicate, maintaining constitutive production in basal conditions. However, when an exaggerated production of some of these mediators is induced as a result of an injury, NA effect is reversed helping to maintain homeostasis. Based on this, we analyzed NA effect on the expression of some well-known pro-inflammatory mediators, namely, inflammatory cytokines. Due to the large number of existing pro-inflammatory cytokines, a selection was made as in the case of the chemokines. Interleukin-1 beta (IL-1β) and TNFα were selected, since these cytokines are known to be produced by astrocytes, and have toxic and trophic actions on neurons [24-26]. As shown in Figure 6, IL-1β mRNA levels were elevated as a result of the incubation of astrocytes with NA, reaching their maximal after 2 hours and decreasing afterwards to control levels. On the other hand, TNFα expression was reduced by NA. However, in the presence of LPS, NA effect was inhibitory for both cytokines (Figure 6B). This effect was also observed when measuring the concentration of TNFα released to the culture medium (Figure 6C).

**Figure 7:**
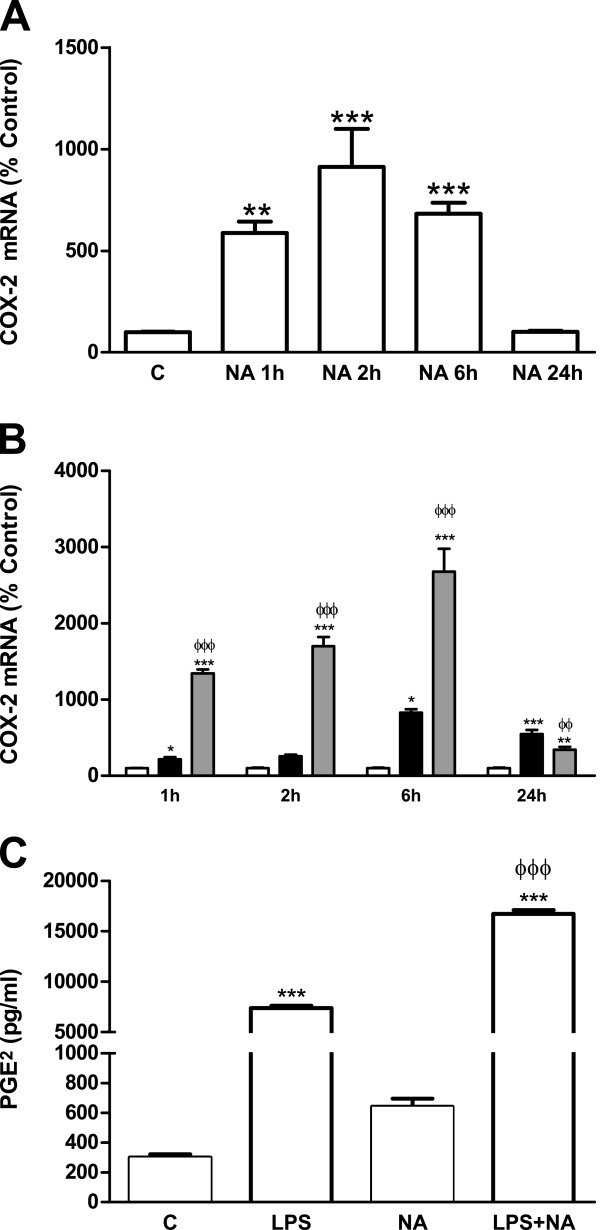
**NA effects on COX-2 and PGE**_**2**_**. (A)** Astrocytes were incubated with control media or NA 10 μM for 1, 2, 6 or 24 hours. RNA was isolated and mRNA levels of COX-2 were determined by RT-PCR. Data are expressed as percentage of control values (set to 100%). **P <0.01 versus control; ***P <0.001 versus control. Data are means ± SE of n = 8 replicates per group. **(B)** Astrocytes were incubated with control media (white columns), LPS 0.1 μg/ml (black columns) or LPS and NA 10 μM (gray columns) for 1, 2, 6 or 24 hours. RNA was isolated and COX-2 mRNA levels determined by RT-PCR. Data are expressed as percentage of control values (set to 100%). *P <0.05 versus control; **P <0.01 versus control; ***P <0.001 versus control; ^ΦΦ^*P* <0.01 versus LPS; ^ΦΦΦ^*P* <0.001 versus LPS. Data are means ± SE of n = 8 replicates per group. **(C)** Astrocytes were incubated with control media, LPS 0.1 μg/ml, NA 10 μM, or LPS and NA for 24 hours. PGE_2_ levels in the media were assessed by EIA. ***P <0.001 versus control; ^ΦΦΦ^*P* <0.001 versus LPS 0.1 μg/ml. Data are means ± SE of n = 8 replicates per group. C, control; COX-2, cyclooxygenase-2; EIA, enzyme immunoassay; LPS, lipopolysaccharide; NA, noradrenaline; PGE_2_, prostaglandin E_2_; RT-PCR, reverse transcription polymerase chain reaction; SE, standard error.

Together with chemokines and cytokines, certain enzymes are key regulators of the inflammatory response. Nitric oxide synthase 2, inducible (NOS2) is known to be inhibited by NA, contributing to the anti-inflammatory and neuroprotective actions of NA [20,27]. Another enzyme which catalyzes the production of multiple products, many of which have inflammatory potential, is cyclooxygenase-2 (COX-2). As shown in Figure 7, NA treatment elevated COX-2 mRNA and in the presence of LPS its effect was amplified (Figure 7B).

The pattern found for COX-2 production is different from those found for all the other proteins analyzed. This may be related to the differences in regulatory mechanisms. Based on the data, we decided to examine the effects of LPS and NA on COX-2 pathways further, particularly PGE_2_. To this end, PGE_2_ concentration in the culture media was measured by EIA and allowed us to observe modifications similar to those found for COX-2 (Figure 7C).

## Discussion

The present study demonstrates the induction of CX3CL1 by NA in astrocytes. Given the neuroprotective actions described for CX3CL1 [28], its regulation by NA could help to explain the mechanisms through which NA protects neurons against different stimuli [29-31]. However, besides its anti-inflammatory and neuroprotective roles, CX3CL1, in its membrane anchored and soluble forms, also functions as a chemoattractant able to activate inflammatory cells [32]. In fact, an exaggerated response of CX3CL1 to certain injuries can lead to irreversible neuronal damage [16-18].

Like CX3CL1, CCL2 is another chemokine known to have certain protective actions in the CNS [33-37], but is also able to potentiate the inflammatory response and cause cell damage [38-40]. Similarly to CX3CL1, we had previously described CCL2 induction by NA in astrocyte cultures [9], as well as in mouse brain cortex astrocytes [41] and its complex regulation by different adrenergic receptors [42]. Both cytokines are expressed by cultured astrocytes and seem to be independently regulated by certain stimuli [43].

However, despite these dual effects of CCL2 and CX3CL1, NA is known to reduce brain inflammation [5,44] and prevent the progression of pathologies with a neuroinflammatory component [45-48]. This led us to hypothesize that in basal conditions, NA may help to maintain CNS levels of different cytokines and chemokines necessary for homeostasis, but under inflammatory conditions, NA prevents an exaggerated production of some of these mediators with neurotoxic potential. In agreement with this, the maximal concentration of CX3CL1 production reached after NA treatment was considerably lower than that observed when LPS was used instead.

Based on our data, the reduction of brain NA levels observed in certain neurodegenerative pathologies, such as Alzheimer’s [6] and Parkinson’s [49] diseases, could be the reason for the parallel loss of the constitutive production of these chemokines. In fact, there is a decreased production of CX3CL1 in the cortex and hippocampus of transgenic amyloid precursor protein (APP) mice together with an elevated accumulation of amyloid beta (Aβ) [50]. This could be in agreement with those studies where the reduction of NA production either by depletion of locus coeruleus neurons [51] or by genetic alterations [52] aggravated the neurological damage in models of Alzheimer’s disease.

While NA effects on chemokines production seem to be mediated through its interaction with adrenergic receptors [9,42], the presence of major histocompatibility complex class II molecules or cluster of differentiation 14 (CD14) proteins detected on the surface of stimulated astrocytes [53,54], suggests that the promoter requirements and pathways leading to the production of the different chemokines and cytokines analyzed may be different for both types of stimuli.

The PCR analysis of CCL7, CCL12 and CXCL16 reveals an inhibitory effect of NA in the presence of LPS for all of them, independently of NA effect in the absence of other stimulus. A similar pattern is observed for CCL6 concentration in the culture medium, while its mRNA levels are reduced by LPS. This indicates that in this case some post-transcriptional alterations are involved, resulting in a regulation similar to that observed for the other cytokines and chemokines analyzed.

Our results suggest that in the presence of an inflammatory stimulus, such as LPS, the actions of NA with respect to the expression of certain cytokines seem to be oriented towards the reversion of LPS alterations, independently of the changes NA may produce in the absence of other stimuli. This possibility constitutes an interesting new research subject, since the modification of NA actions on astrocytes are probably due to transformations caused by the activation of these cells as a response to an injury or any threat to homeostasis [55].

The induction by LPS of IL-1β and TNFα is a well-known response of astrocytes [56,57], and the inhibition caused by NA is in agreement with its neuroprotective effects due to the pro-inflammatory nature of these cytokines [58,59]. On the other hand, it was more surprising to detect the induction of IL-1β by NA. Nevertheless, this could be in agreement with the above mentioned hypothesis, since this cytokine has been described to help protect neurons against certain types of injuries [24,26].

NA effects on COX-2 production have been previously analyzed in microglia by Schlachetzki *et al*. [60]. They also observed an induction by NA that was potentiated by LPS. Similarly to what we found for astrocytes, their work also describes the release of PGE_2_ by microglia in response to LPS or NA and the boosting of this effect by the combination of both treatments. Considering the involvement of PGE_2_ in the development of neuroinflammation [61], this effect of NA seems contradictory with its neuroprotective actions. However, PGE_2_ is another mediator for whom neuroprotective actions have also been discovered [62-65]. While this could help to explain our results, the additive actions of LPS and NA on COX-2 and PGE_2_ expression, eliminate the possibility of a simplistic explanation according to which NA reverses the changes caused by an inflammatory stimulus on astrocytes. This confirms the complex nature of NA mechanisms of action and, in particular, reveals the need to study PGE_2_ interactions with neurons and if the presence of NA also modulates the response to PGE_2_.

## Conclusions

While NA neuroprotective actions are largely confirmed by different studies, its mechanisms of action are not well-known. MCP-1 and CX3CL1 induction could help to explain some of NA effects due to their ability to prevent neuronal damage under diverse conditions. However, such effect could be reversed when these and other mediators are produced in an exaggerated/uncontrolled manner. The data presented here indicate that NA may help to maintain the production of certain chemokines, while preventing their overproduction and subsequent toxicity. Further investigations of *in vivo* models may help confirm this hypothesis, extend the understanding of NA neuroprotective role and hopefully facilitate the development of NA-based therapies for neurodegenerative diseases.

## Abbreviations

ANOVA: Analysis of variance; APP: Amyloid precursor protein; Aβ: Amyloid beta; CCL12: Chemokine (C-C motif) ligand 12; CCL2: Chemokine (C-C motif) ligand 2; CCL6: Chemokine (C-C motif) ligand 6; CCL7: Chemokine (C-C motif) ligand 7; CCR2: C-C chemokine receptor type 2; CD14: Cluster of differentiation 14; CNS: Central nervous system; COX-2: Cyclooxygenase-2; CX3CL1: Chemokine (C-X3-C motif) ligand 1; CXCL16: Chemokine (C-X-C motif) ligand 16; DMEM: Dulbecco’s modified Eagle’s medium; EIA: Enzyme immunoassay; ELISA: Enzyme-linked immunosorbent assay; EU: Endotoxin units; GAPDH: Glyceraldehyde 3-phosphate dehydrogenase; GFAP: Glial fibrillary acidic protein; IFNγ: Interferon gamma; IL-1β: Interleukin-1 beta; LPS: Lipopolysaccharide; MCP-1: Monocyte chemotactic protein-1; MCP-3: Monocyte chemotactic protein-3; MCP-5: Monocyte chemotactic protein-5; NA: Noradrenaline; NOS2: Nitric oxide synthase 2 inducible; PCR: Polymerase chain reaction; PGE2: Prostaglandin E_2_; qPCR: Real-time polymerase chain reaction; qRT-PCR: Real-time reverse transcription polymerase chain reaction; RT-PCR: Reverse transcription polymerase chain reaction; SCYB16: Small inducible cytokine subfamily B member 16; SE: Standard error; TNFα: Tumor necrosis factor alpha.

## Competing interests

The authors declare that they have no competing interests.

## Authors’ contributions

AH carried out the cell assays, RT-PCR, data acquisition and helped to draft the manuscript. JC, BG and JL contributed to analysis and interpretation of data, and drafted and critically revised the manuscript. JM performed cell cultures and treatments, conceived the study, participated in its design and coordination, and helped to draft the manuscript. All authors read and approved the final manuscript.
